# Novel grasp-and-snare technique for efficient endoscopic necrosectomy of walled-off necrosis

**DOI:** 10.1055/a-2325-5739

**Published:** 2024-06-07

**Authors:** Haruka Toyonaga, Tsuyoshi Hayashi, Kazuki Hama, Toshifumi Kin, Masayo Motoya, Kuniyuki Takahashi, Akio Katanuma

**Affiliations:** 1 37009 Gastroenterology, Teine Keijinkai Hospital, Sapporo, Japan

Walled-off necrosis (WON) is a significant local complication of severe pancreatitis. Despite the utilization of endoscopic ultrasound-guided drainage, if necrotic debris accumulates within the WON and infection remains uncontrolled, direct endoscopic necrosectomy (DEN) is performed using auxiliary instruments such as forceps or snares 1 2 . Conventional techniques often involve prolonged and multiple sessions because of their limited ability to remove necrotic material, leading to a substantial patient burden. Here we present an efficient adaptation of the two-channel method 3 , commonly utilized for endoscopic mucosal resection ( Fig. 1 ), for DEN, termed the “grasp-and-snare” technique. The procedure involves the use of a dual-channel endoscope (GIF-2TQ260M; Olympus, Tokyo, Japan), with one channel used to insert forceps for grasping and pulling lesions toward the operator and the other channel used to insert a snare for resecting the lesions. This technique enables simultaneous traction and snaring, rendering DEN safe and efficient ( Fig. 2 , Video 1 ).

**Fig. 1:**
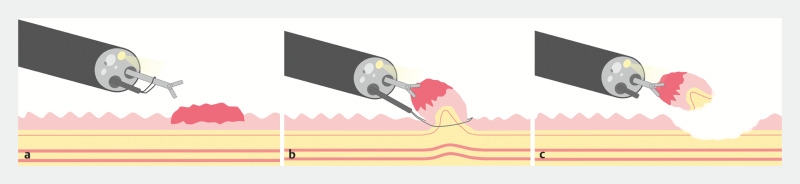
Schematic diagram of the two-channel method for endoscopic mucosal resection using a dual-channel endoscope. **a** The snare is looped around the forceps. **b** The lesion is retracted using forceps, and the snare is applied around the lesion. **c** This technique ensures complete lesion removal and a low risk of damage to the muscular layer due to traction.

**Fig. 2:**
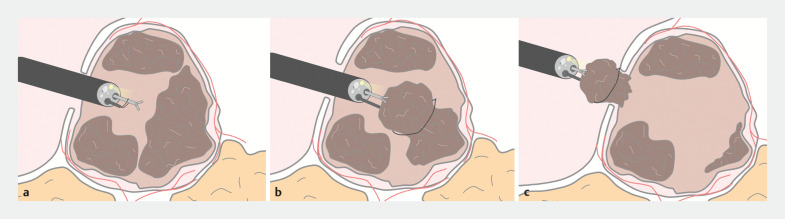
Schematic diagram of the grasp-and-snare technique for necrosectomy in walled-off necrosis (WON). **a** The forceps with the snare looped around are positioned within the WON cavity. **b** The necrotic tissue is grasped and retracted with the forceps, and the snare is applied around the retracted necrotic tissue to detach it from the inner wall of the WON and vessels. **c** The necrotic tissue is removed by pulling on both the forceps and snare. In cases where the necrotic tissue cannot be removed due to fibrosis and no blood vessels were seen in the cavity on prior dynamic computed tomography, the tissue is cut by applying electrical current.

Grasp-and-snare technique is safe and efficient for direct endoscopic necrosectomy (DEN) of walled-off necrosis (WON) following severe pancreatitis. This technique has been adapted from the two-channel method used for endoscopic mucosal resection.Video 1

A 47-year-old woman presented with a massive WON on computed tomography following severe gallstone pancreatitis ( Fig. 3 ), for which conventional DEN proved inadequate for the removal of substantial necrotic material. Prior imaging confirmed the absence of blood flow within the necrotic cavity, endorsing the safety of invasive necrosectomy. The grasp-and-snare technique involved grasping and pulling the necrotic tissue using forceps operated through one channel of the endoscope, followed by precise snaring and removal of the extracted tissue via the other channel ( Fig. 4 ). Any necrotic tissue resistant to tearing could be safely cut using high frequency electrical current (endoCUT Q mode, effect 2.0, VIO3; Erbe Elektromedizin, Tübingen, Germany).

**Fig. 3:**
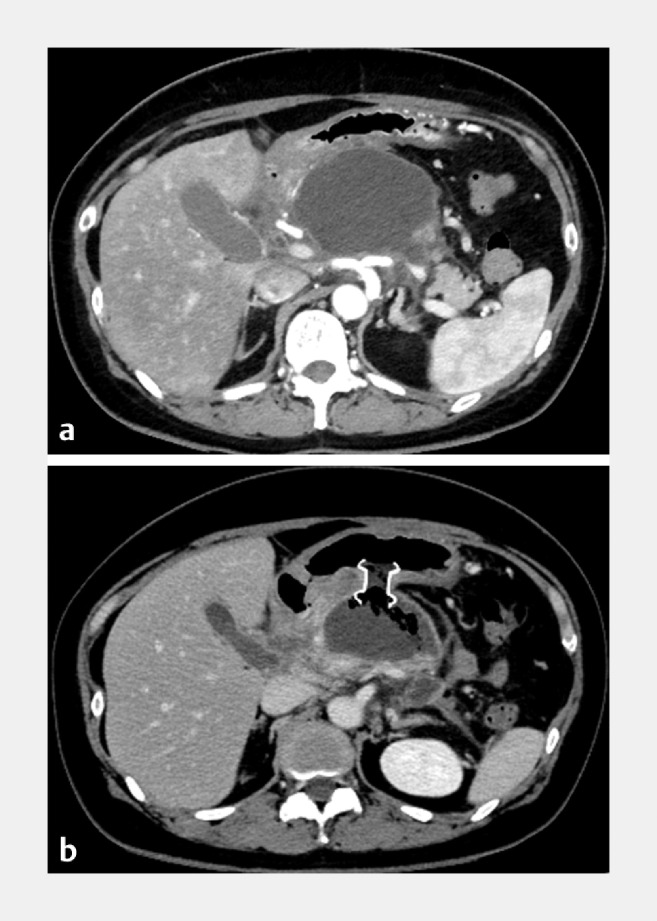
Dynamic computed tomography images of walled-off necrosis (WON). **a** Before endoscopic ultrasound-guided cyst drainage, the pancreatic body was completely necrotic and its head and tail were disconnected. The lumen of the WON was filled with a large amount of necrotic tissue, and no blood vessels were identified. **b** A large amount of necrotic tissue remained after drainage with a lumen-apposing metal stent.

**Fig. 4:**
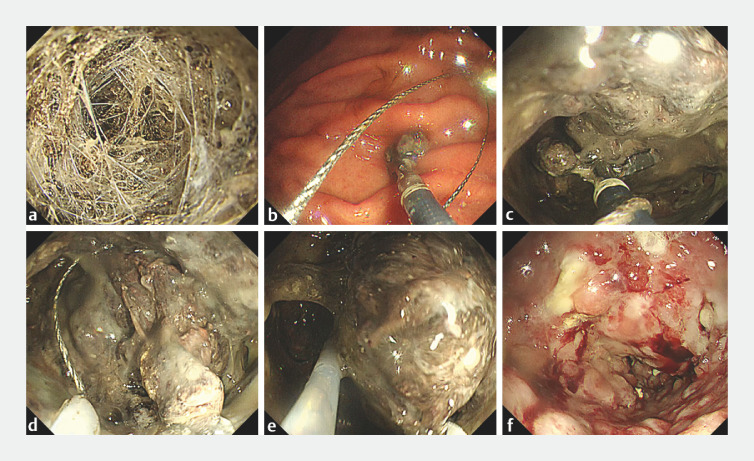
Endoscopic images of direct endoscopic necrosectomy (DEN) for walled-off necrosis (WON) using the grasp-and-snare technique. **a** The necrotic tissue was hard and adherent and contained a large amount of fibrous components. **b** Prior to entering the WON cavity, the snare was looped around the forceps. **c** Upon entering the WON, the hard necrotic tissue was grasped with the forceps and retracted toward the operator. **d, e** The necrotic tissue retracted by the forceps was grasped with the snare ( **d** ) and removed using the forceps and snare ( **e** ). In cases where the tissue cannot be torn due to its hard consistency and the absence of vascular structures has been confirmed on computed tomography, electrical current can be used to safely cut the tissue retracted by the forceps. **f** Although the WON cavity was filled with large amounts of hard necrotic tissue, the grasp-and-snare technique efficiently and safely removed the necrotic tissue.

The grasp-and-snare technique can be used to safely remove a larger volume of necrotic debris in fewer attempts, potentially reducing the overall treatment time and patient burden.

Endoscopy_UCTN_Code_TTT_1AS_2AJ

## Notes

Endoscopy E-Videos https://eref.thieme.de/e-videos
*E-Videos* is an open access online section of the journal *Endoscopy* , reporting on interesting cases and new techniques in gastroenterological endoscopy. All papers include a high-quality video and are published with a Creative Commons CC-BY license. Endoscopy E-Videos qualify for HINARI discounts and waivers and eligibility is automatically checked during the submission process. We grant 100% waivers to articles whose corresponding authors are based in Group A countries and 50% waivers to those who are based in Group B countries as classified by Research4Life (see: https://www.research4life.org/access/eligibility/ ). This section has its own submission website at https://mc.manuscriptcentral.com/e-videos .
